# A Model-Based Clustering Method for Genomic Structural Variant Prediction and Genotyping Using Paired-End Sequencing Data

**DOI:** 10.1371/journal.pone.0052881

**Published:** 2012-12-27

**Authors:** Matthew Hayes, Yoon Soo Pyon, Jing Li

**Affiliations:** Department of Electrical Engineering and Computer Science, Case Western Reserve University, Cleveland, Ohio, United States of America; Wayne State University, United States of America

## Abstract

Structural variation (SV) has been reported to be associated with numerous diseases such as cancer. With the advent of next generation sequencing (NGS) technologies, various types of SV can be potentially identified. We propose a model based clustering approach utilizing a set of features defined for each type of SV events. Our method, termed SVMiner, not only provides a probability score for each candidate, but also predicts the heterozygosity of genomic deletions. Extensive experiments on genome-wide deep sequencing data have demonstrated that SVMiner is robust against the variability of a single cluster feature, and it significantly outperforms several commonly used SV detection programs. SVMiner can be downloaded from http://cbc.case.edu/svminer/.

## Introduction

Genomic structural variation refers to the rearrangement, duplication, deletion, or insertion of DNAs in the human genome [1]–[4]. Recent studies have shown that in addition to single-nucleotide polymorphisms (SNPs), genomic structural alterations may be responsible for much of the phenotypic variation that currently exists amongst human populations. These genomic structural variants are ubiquitous and some are likely responsible for increased susceptibility to diseases such as cancer [5]–[7], as well as other conditions such as obesity and infertility [7], [8]. Given the impact of structural variation on individuals, locating and mapping these regions can provide better understanding of the genetic mechanisms that define phenotypic differences and those that can potentially trigger diseases and disorders [4]. Among the platforms used to detect structural variation are array comparative genomic hybridization (aCGH), SNP genotyping arrays, and high throughput next generation sequencing (NGS). Sequencing technologies (e.g., Illumina Genome Analyzer, ABI SOLiD, and Roche's 454) not only allow for genome-wide screening of structural variants at the nucleotide resolution [4], [9], [10], but also allow for detection of different types of structural variation (SV) events using paired-end sequencing (for reviews, see Refs [11]–[13]).

**Figure 1:**
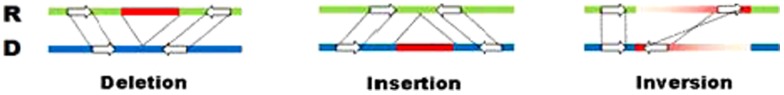
Structural variants and the read pairs that support them. Pairs from a donor genome “D” have distinct characteristics when mapped to a reference genome “R” for different types of structure variants.

To detect structural variants using paired-end NGS data, millions of paired sequence reads of roughly known lengths are first generated from a donor genome. For a given read pair, the distance from a read to its mate is known as the *insert* size, and these paired reads are computationally mapped to specific locations on a reference genome. When a pair of reads does not overlap with any structural variant, the mapped distance between them should approximately be the same as the library insert size and should have a different orientation. On the other hand, when the pair *does* overlap a structural variant, the mapping distance will deviate from the insert size or its orientation will be different from that of a normal pair of reads, or both, depending on the types of structural variants. One advantage of using paired-end sequence data is that it allows for detection of many types of SVs. Read pairs with normally mapped distances and correct orientation are known as *concordant* pairs, else the pairs are *discordant* and suggest a possible variant. The relationships between discordant pairs and three types of possible structural variants are shown in Figure 1.

Most existing computational approaches were developed based on mapping distance and/or orientation for high coverage data from a single genome [11]–[13]. A few groups [14]–[16] have provided the first studies in which a human genome was sequenced using next generation technology, and called structural and other genetic variation, mainly using their in-house tools. In addition, many algorithms have been proposed recently [12]. Hormozdiari *et al.* [17] presented three algorithms for detecting structural variation in a donor sequence using NGS technology based on two different formulations. Their first formulation is called the “Maximum Parsimony Structural Variation” (MPSV) problem, the goal of which is to compute a unique mapping for each discordant read pair in the reference genome such that the total number of implied structural variants is minimized. Two algorithms (named VariationHunter_unweighted and VariationHunter_weighted, or VHU and VHW for short) were developed, both of which were based on an approximation algorithm for the Set-Cover problem. Their second formulation aims to calculate the probability of each event and an iterative algorithm was proposed (named VariationHunter_Probabilistic or VHP). They further developed their algorithms for transposons [18]. Lee *et al.* [19] presented an algorithm called “MoDIL”, which primarily focuses on detecting insertions and deletions of small sizes ranging from 20 to 50 base pairs (bps). The main idea is to compare the distribution of insert sizes in the library to the distribution of the observed mapped distances, utilizing an iterative Kolmogorov-Smirnov (KS) test. MoDIL can determine heterozygosity of deletions by observing the distribution of mapped distances within a variant cluster, though it does not consider read depth for this process. Chen *et al.* [20] presented two algorithms for detecting structural variation using NGS technology. The first algorithm, BreakDancerMax, can potentially detect large deletions, large insertions, inversions, and translocations. Their second algorithm, BreakDancerMini, focuses on small indels that are between 10–100 base pairs in length. BreakDancerMax identifies SV events by grouping together overlapping discordant pairs of a certain type, and the region is called as a structural variant based on a confidence score that is a function of the number of discordant pairs, the coverage of the reads, and the size of the region defined by the discordant pairs. The BreakDancerMini algorithm, which is in principle similar to the MoDIL algorithm [19], locates small indels by dividing the genome into non overlapping windows and comparing the distribution of mapped distances for read pairs in the window with the distribution of mapped distances in the entire genome using the KS test.

However, in most cases, it is not enough to call a structural variant based only on these two characteristics (*i.e.*, mapping distance and orientation). First of all, the distribution of the insert size is usually estimated. In many cases, it is difficult to distinguish whether a deviation of mapping distance from the mean insert size is due to a structural variant or due to the fact that the segment of this particular pair happens to be far from/close to the mean. In addition, sequencing errors and mapping uncertainties all add to the complexity of identifying structural variants. The aforementioned challenges are what motivate existing approaches [17], [19], [20] that address this problem.

In this paper, we present a model-based approach for SV detection named SVMiner. SVMiner uses as input the mapping of DNA paired-end reads to a reference sequence, which can be obtained using existing mapping tools (e.g., MAQ [21] and BWA [22]). It consists of four steps, namely, a) classification of paired-end reads into different categories according to their separation distances and orientations; b) candidate SV generation by grouping overlapped read pairs of the same type and similar lengths that are in close proximity; c) feature collection based on types of SV candidates; and d) a model-based clustering approach to separate candidates into final predicted events or normal regions. SVMiner has three distinct features: 1) in addition to separation distances and orientations, SVMiner automatically collects new features for different types of events; 2) SVMiner further utilizes these new features for classification and can predict heterozygosity for deletion events; 3) the model-based clustering approach also reports membership probabilities, which could be interpreted as confidence scores. We implement the approach and apply it on two recently generated datasets [14] as well as a simulated dataset. In addition, we also apply the method to a dataset of the same individual, but with a longer read length. In our experiments, we investigate the effect of mapping tool, insert size, read length and assess its capability in reporting heterozygous deletions and event membership probabilities. We compare our results with those from the original papers, experimentally validated events of the same sample using a different technology [23], results from the 1000 genome project [3], [24], as well as prediction results of three popular programs for detecting SV using paired-end reads: BreakDancerMax [20], VariationHunter [17], and MoDIL [19]. Although the ground truth of structure variant events is generally not available for real data, we construct several highly reliable event sets based on 1) the reported results from the original papers; 2) predictions that overlap with events from the 1000 genome project; 3) events predicted by at least two independent algorithms. Based on these reliable event sets, SVMiner significantly outperforms all the three approaches in detecting deletions. Furthermore, it achieves much higher accuracy than MoDIL in predicting heterozygous deletions. Simulation results on deletions also show that SVMiner has the best overall performance. For inversions, the overlaps among different programs are low, indicating it is much hard to detect inversions for all the programs tested.

## Results

### Outline of the algorithm

SVMiner takes as input a set of alignment results and outputs a list of likely SV events with probability scores. It is worth mentioning that there exist many different mapping algorithms with different characteristics, and in principle, mapping results from any algorithm can be used in our approach. In our implementation, SVMiner can take alignment results from MAQ [21] and BWA [22], or any other mapping tools that support BAM format. As previously stated, SVMiner consists of four steps. Firstly, the insert size and its standard deviation will be estimated based on data and each pair will be classified based on either 1) its separation distance relative to the insert size or 2) its orientation. Table 1 summarizes all different types of read pairs. In this study, we only focus on deletion and inversion events, thus only discordant pairs for deletions and inversions will be studied. Secondly, SVMiner generates SV candidates by grouping overlapped discordant read pairs of the same type and similar lengths that are in close proximity. Thirdly, for each type of candidate SVs, we define a feature space to be used in step 4 by our model-based clustering method. Finally, we use a model-based clustering approach to separate candidates into predicted variants or predicted regions of no variation. The first two steps of our algorithm are shared by many existing approaches. Our major contribution is to infer variant heterozygosity, to develop a procedure to automatically collect different features for different types of SV events (step 3) and to assign membership probabilities for each candidate SV (step 4). Feature spaces are different for different types of events, but mainly based on the number of discordant pairs, the coverage of normal pairs, and the number of singletons (i.e., only one read of a pair being mapped to the reference), with the aim to distinguish real events from false ones and to distinguish heterozygous events from homozygous ones. The exact definitions of features for deletions and inversions will be discussed in the section of Materials & Method.

**Table 1:** Classification of read pairs.

	d<μ-x*σ	μ-x*σ≤d≤μ+x*σ	μ+x*σ≤d	Different Chromosomes
**FR**	I	N	D, Tra	Tre
**RF**	Ta	Ta	Ta, Tra	Tre
**FF**	Iv			C
**RR**	Iv			C

The relationship between paired-end reads and structural variants according to a partition based on their separation distances and orientations. The meanings of symbols: F: forward read; R: reverse read; d: separation distance; μ: insert size (mean); σ: standard deviation; *x*: a user-defined parameter; N: normal; D: deletion; I: insertion; Iv: inversion; Ta: tandem duplication; Tra: intrachromosomal translocation; Tre: interchromosomal translocation; C: other complex structure event.

Once a feature set is defined and collected for each candidate SV, each candidate is viewed as one observation generated from a mixture distribution, which can then be predicted as either a true variant, or a false event (i.e., region of no variation). For a predicted true event, there are also two possibilities: a homozygous event where both homolog chromosomes/haplotypes bear the same type of SV, or a heterozygous event where only one chromosome/haplotype has the event. We construct features such that for different types of events (homozygous, heterozygous and normal), they exhibit different characteristics and represent different mixture components. Therefore, in our framework, the number of mixture components, G, is naturally defined as 3. However, for inversions, the data is so noisy that one cannot really distinguish heterozygous events from homozygous events in practice. Therefore, we only infer heterozygosity for deletions. For inversions, we consider a candidate SV can be predicted as either a true event or a normal region (i.e., G = 2). For our model-based approach, we adopt the one described in [25]. More details of the approach can be found in the Materials & Methods section.

### Experimental design

We evaluated our method on three real datasets as well as on simulated datasets for both deletion events and inversion events, and mainly compared our approach with three existing algorithms, *i.e.*, MoDIL, BreakDancer, and VariationHunter. The three real datasets are an African male (NA18507) whole genome data with read lengths of 36–41 bps and an insert size of 200 bps [14] (dataset I), the same sample (NA18507) with different read lengths (∼100 bps) and different insert size (∼500 bps) (dataset II), and a Caucasian female (NA07340) chromosome X data with read lengths of 30–35 bp and an insert length of 130 bps [14] (dataset III). The reads for these datasets were generated using the Illumina Genome Analyzer II, and downloaded from the NCBI short read archive (NA18507) and European Bioinformatics Institute (NA07340). For dataset III, the original study [14] also predicted homozygous and heterozygous deletions by manual examinations, which therefore was used to assess capability of SVMiner in detecting heterozygous deletions. The simulated datasets were generated following the procedure presented in the Materials & Methods section and were used in testing the functionalities of the software and in comparing with other tools. For deletions, we evaluated the performance of SVMiner by examining its results using different mapping tools, the effect of using membership probabilities as a reliability measure, its prediction on heterozygosity, and the effect of different insert sizes and read lengths. We compared its results with the results reported in the original papers as well as results reported from the three existing programs, namely, BreakDancer, VariationHunter and MoDIL. The three programs were chosen not only because of their functionality, popularity and availability, but also because they have been used in studying dataset I. Not all programs were tested on all datasets or all parameter settings, because 1) programs may have some special requirements, which makes some experiments unnecessary or infeasible (*e.g.*, VariationHunter uses its own mapping tool; some other programs do not distinguish heterozygous deletions from homozygous ones); 2) due to the huge amount of sequence data, computational resources limit the number of possible combinations. When comparing results from two programs on a dataset or comparing predictions with events in the reliable sets, we examine the overlaps between corresponding events. Two events are considered the same one if they have at least 50% reciprocal overlap with each other.

### Results using different alignment tools

Structural variation prediction critically depends on the quality of mapping results. Therefore, we first tested SVMiner on dataset I using two alignment tools: BWA and MAQ. MAQ provides three types of mapping quality scores for paired-end reads: a single-end mapping quality score for each read, a mapping quality score (Q) for each pair, and an alternative mapping quality score (AltQ) for each pair [21]. The single-end score of each read is a measure of mapping quality of the read, defined as 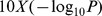, where *P* is probability that the read is erroneously mapped. AltQ is defined as the minimum of the two single-end mapping quality scores. The mapping quality score Q for a pair of reads is defined differently for different types of read pairs. For properly mapped pairs (i.e., uniquely mapped pairs with correct orientations and a proper mapping distance), it is the summation of the single-end mapping quality scores of both ends. For all other cases (including discordant pairs or pairs with multiple mapping positions), it is defined as the minimum of the two single-end mapping quality scores, which is the same as AltQ in such a case. BWA only provides one mapping quality score for each pair, which is more or less close to the definition of mapping quality score of each pair in MAQ. Although the quality scores from the two programs are conceptually related, they do not always correlate well (Figure S1). To retain highly reliable mapped reads, a threshold is normally used to filter out low quality reads. We first investigated the effect of both Q and AltQ for MAQ, and the mapping quality score for BWA on mapping results.

In terms of mapping results of the two programs, around 96.5% (MAQ) and 98.1% (BWA) reads out of total 3.77B reads have been mapped to the genome (Table 2). Among them, BWA has ∼72.4% of mapped read pairs with quality score > = 30; MAQ has about 76.3% read pairs with mapping quality score Q> = 30. However, if one uses the alternative quality score of MAQ, only 49.5% mapped read pairs have AltQ> = 30. The number of discordant pairs also varies significantly (from 414 K to 797 K) for these thresholds (Table 2). In addition to algorithmic differences, another possible reason is that the two mapping tools have different error models in calculating mapping probabilities, and the two quality scores cannot easily map to each other. As noted by Li et al. [22], the actual quality score of MAQ tends to be underestimated, while the quality score of BWA tends to be overestimated, although the concept of the mapping quality scores of both alignment tools is the same. Therefore, using different mapping tools and different (or even the same) thresholds for quality control, the mapped results, which are also the inputs to our program SVMiner, are different. It is expected that the results of SVMiner will also be different. Nevertheless, a significant majority of the events are still the same. For example, 4384 deletions are in common between the results using BWA with quality scores≥30 (6279 deletions in total) and the results using MAQ with quality scores Q≥30 (5209 deletions in total). For MAQ with alternative mapping quality score AltQ≥30, our approach identified 5251 deletions. The numbers of total candidate events and the numbers of predicted homozygous deletions, heterozygous deletions and normal events based on the three thresholds of the two alignment tools are shown in Table 2. Although the numbers of initial candidates based on MAQ with two different thresholds differ much, the numbers of final predicted events are close (Table 2). However, the numbers of homozygous/heterozygous events are quite different (Table 2). This is mainly because when using the mapping quality score Q, more concordant read pairs, which may not be of high quality and would be removed when using AltQ, will be retained, which result in more concordant pairs across the genome, including candidate regions. SVMiner therefore predicted more heterozygous events. The results of SVMiner are inevitably affected by the results of alignment tools. Nevertheless, the inconsistency mostly happens for events with weak signals. From this point of view, SV prediction approaches that can provide reliability scores, such as SVMiner proposed here, are desirable, because one can potentially use the reliability score to filter low quality events. We decided to use mapping results from BWA for the remaining experiments, mainly because of its efficiency.

**Table 2:** Mapping results and SV calling results of SVMiner using different alignment tools and different parameter thresholds.

	# reads	# mapped reads	# mapped reads (score≥30)	# discordant reads for deletions (shared with BWA)	Ave depth of normal pairs in candidates	Ave # of discordant pairs in candidates	# of candidates, homozygous, heterozygous, normal events
BWA	3.77b	3.697b	2.677b	0.671 m	10.53	21.17	8627, 3505, 2774, 2348
MAQ (Q)	3.77b	3.637b	2.774b	0.797 m (0.457 m)	12.31	19.31	7785, 2361, 2848, 2576
MAQ (AltQ)	3.77b	3.637b	1.802b	0.414 m (0.346 m)	5.72	21.66	6464, 3372, 1879, 1211

### Membership probabilities as reliability scores

**Figure 2:**
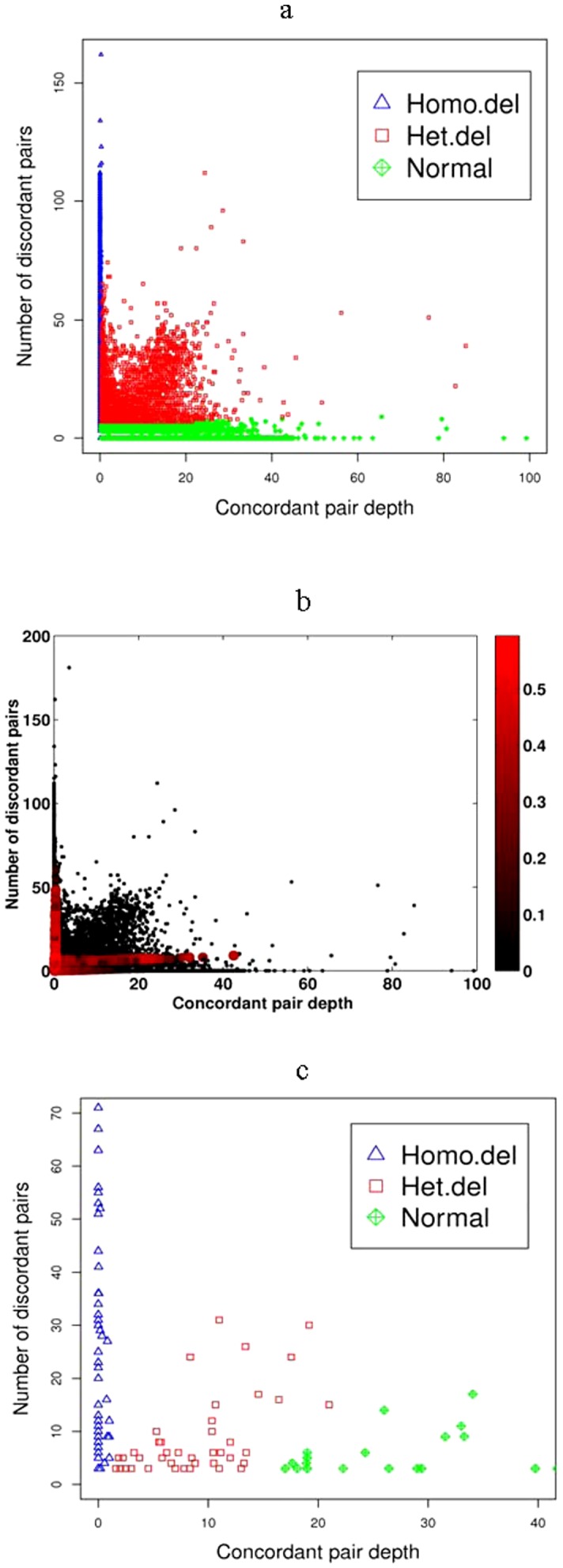
Predicted deletion events and their uncertainty scores. (a) The clustering results of the candidate deletions of dataset I (NA18507). (b) Plot of uncertainty for predicted results from the same dataset. Uncertainty is defined as 1−*z*, where *z* is the greatest of the class membership probabilities after clustering. The larger, darker circles indicate lower membership probabilities for the given data point. The highest degrees of uncertainties are around cluster boundaries and around the origin where the data features are not prominent enough to allow for a more confident prediction. Data points with prominent features (i.e. high concordant pair depth and/or high number of discordant pairs) are generally classified with high confidence. (c) The clustering results of the candidate deletions of dataset III (chromosome X of NA07340).

The membership probabilities provided by SVMiner for each predicted event can be used as reliability scores to rank and filter predicted events. Figure 2A displays classification results on dataset I and Figure 2B shows the corresponding prediction uncertainty score of each data point, which is defined as one minus the maximum of the three membership probabilities. The general shape of the clusters suggests that SVMiner results are generally robust to the variability amongst feature values within the same class. Consequently, classification results tend to be robust against differing levels of sequence coverage. It is also apparent that a candidate variant with accentuated features is very likely to be predicted as a true variant. This can be seen from the uncertainty plot. Points of higher uncertainty tend to accumulate near the cluster boundaries, especially near the origin. Because data points near the origin have low concordant pair depths and low numbers of discordant pairs, they have reasonably moderate probabilities of being *any* event. In particular, it is difficult to separate heterozygous predictions from normal predictions in these regions (Figure S2). Otherwise, Figure S2 shows that the distributions of features of the three different types of events generally agree with expectations. We further examined the membership probability distributions of predicted normal events. In general, the membership probabilities for data points with weak feature values are lower than those with prominent features (Figure S3). This suggests that one can utilize our reported membership probabilities in distinguishing high quality events from events with high uncertainly. A simple threshold on the maximum membership probability can be adopted to return only events with high confidence.

### Genotyping results

**Figure 3:**
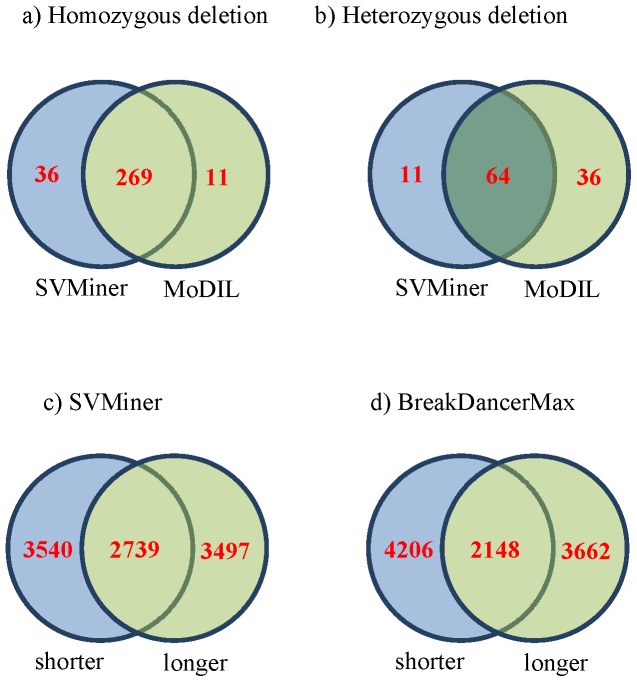
Overlaps between SVMiner and MoDIL, and between SVMiner and BreakDancerMax on sample NA18507. Top: summary of overlaps between SVMiner and MoDIL for homozygous deletions (a) and heterozygous deletions (b). Bottom: overlaps between shorter read data and longer read data for SVMiner (c) and BreakDancerMax (d).

Among all the other methods considered, MoDIL [19] is the only tool that can predict heterozygous deletions. However, MoDIL and SVMiner are not directly comparable in the sense that MoDIL was originally designed to detect small to medium sized deletions. Nevertheless, to evaluate the performance of SVMiner in predicting heterozygous deletions, we compare the results of SVMiner and MoDIL on three different datasets: genome-wide data of specimen NA18507 (dataset I), chromosome X data of specimen NA07340 (dataset III) and a simulated dataset. For dataset I, we directly obtained the results from the authors of MoDIL (who used mrFast as their mapping tool). Because MoDIL focused mostly on detecting small to medium size indels (20∼50 bps), only a small portion (6.3%) of their 10,397 reported deletions with high confidence have sizes greater than 50 bps and may directly comparable with our results. Among them, 380 deletions called by MoDIL overlap (50% reciprocal) with events predicted by SVMiner. Figure 3 (top) illustrates the number of overlaps of two different types of deletion events. Among the overlapped events, results show the two programs have a high degree of consistency. For example, for homozygous deletions, 269 common events were found by SVMiner (total 305 predictions) and MoDIL (total 280 predictions). For heterozygous deletions, 64 common events were found by SVMiner (total 75 predictions) and MoDIL (total 100 predictions). SVMiner and MoDIL had a higher degree of mutual overlap for homozygous deletions as compared to heterozygous deletions, which is expected because heterozygous events are more difficult to distinguish from normal events.

One should notice that MoDIL may not be able to precisely predict the two breakpoints of an event. In the result file of MoDIL, both the size of an event and its start/end positions (i.e., breakpoints) were given. However, the distance between the two breakpoints of an event in general is much greater than the event size, which may affect values of those features, based on which MoDIL made its predictions. We examined some of the inconsistent calls by SVMiner and MoDIL, they often disagree on the two breakpoints. Figure S4A shows an example of an event called by SVMiner as a “homozygous deletion”, but a “heterozygous deletion” by MoDIL. According to the breakpoints of SVMiner (dashed lines), there are apparently no concordant pairs within the deletion region, therefore, it concluded that this event is a homozygous deletion. However, according to the predicted breakpoints of MoDIL (dotted lines), this region included enough concordant pairs for the program to predict this event as a heterozygous deletion (Figure S4B). Another possible reason of inconsistency might be due to the fact that two programs had used different mapping tools, which is known to affect the calling results as shown earlier.

Dataset III was originally studied by Bentley *et al.* [14] and the authors manually separated the predicted 77 deletion events into 49 homozygous and 28 heterozygous deletions, which should be of high quality and therefore were regarded as the “ground truth” in comparing SVMiner and MoDIL in this study. Running both programs on this dataset, SVMiner predicted 55 homozygous deletions and 42 heterozygous deletions (Figure 2C), while MoDIL predicted 12 homozygous deletions and 55 heterozygous deletions. A predicted event is treated as a true positive if the region has at least 50% reciprocal overlap with one of Bentley's events. Although 30 out of 67 predictions made by MoDIL overlap with true events, the number of correctly predicted genotypes is very low, with 5 correctly predicted homozygous genotypes and 9 correctly predicted heterozygous genotypes. In contrast, 69 out of 97 predictions made by SVMiner overlap with the true events. In particular, SVMiner correctly predicted 41 homozygous events and 21 heterozygous events. Overall, SVMiner significantly outperformed MoDIL on this dataset (Table 3). The F_scores of SVMiner range from 0.60 to 0.79 when evaluating genotype predictions separately or jointly, however, the F_scores of MoDIL range from 0.16 to 0.42.

**Table 3:** Comparison of SVMiner and MoDIL on two datasets.

Combined							
Data	# real events	Method	# predictions	# true positive	precision	recall	F-score
X chromosome	77	SVMiner	97	69	0.71	0.90	0.79
X chromosome	77	MoDIL	67	30	0.45	0.39	0.42
Simulated	190	SVMiner	153	153	1	0.81	0.89
Simulated	190	MoDIL	1123	137	0.12	0.72	0.21

Homozygous							
Data	# real events	Method	# predictions	# true positive	precision	recall	F-score
X chromosome	49	SVMiner	55	41	0.75	0.84	0.79
X chromosome	49	MoDIL	12	5	0.42	0.10	0.16
Simulated	94	SVMiner	77	75	0.97	0.80	0.88
Simulated	94	MoDIL	3*	3	1.00	0.03	0.06

Heterozygous							
Data	# real events	Method	# predictions	# true positive	precision	recall	F-score
X chromosome	28	SVMiner	42	21	0.5	0.75	0.6
X chromosome	28	MoDIL	55	9	0.16	0.32	0.22
Simulated	96	SVMiner	76	75	0.99	0.78	0.87
Simulated	96	MoDIL	134*	73	0.54	0.76	0.63

* For MoDIL, only 137 high confident ones that have overlaps with real events were counted when considering homozygous/heterozygous predictions, its F_score would be extremely low if all predicted events considered.

In addition to these two real datasets, we also compared SVMiner and MoDIL on a simulated dataset. The data generation procedure was described in the Materials and Methods section. Briefly, 190 deletions (94 homozygous and 96 heterozygous) of varying sizes (ranging from 100 bps to 75 kbps in length) are embedded in one chromosome. In total, MoDIL identified 1123 deletions, which include many small deletions that are false positives. Among all them, only 137 predictions with high confidence overlapped with the embedded deletions. Among the 137 predictions with high confidence, 3 were predicted as homozygous, and 134 were predicted as heterozygous (Table 3). All 3 homozygous predictions overlapped with embedded homozygous deletions. However, only 73 of the 134 heterozygous deletion predictions overlapped with embedded heterozygous deletions, while the remaining 61 overlapped with embedded homozygous deletions. In contrast, SVMiner predicted a total of 153 deletions, all of which overlapped with embedded deletions, yielding a precision of 100%. In terms of homozygosity, out of the 77 predicted homozygous deletions, 75 of them overlapped with embedded homozygous deletions and the other two overlapped with embedded heterozygous events. For the 76 predicted heterozygous deletions, 75 of them overlapped with embedded heterozygous deletions and the other one overlapped with an embedded homozygous deletion. On this simulated dataset, MoDIL not only predicted many false positives, but also had a great tendency to misclassify true heterozygous deletions as homozygous ones. A precision and recall comparison of SVMiner to MoDIL can be found in Table 3.

### Read length and insert size

**Figure 4:**
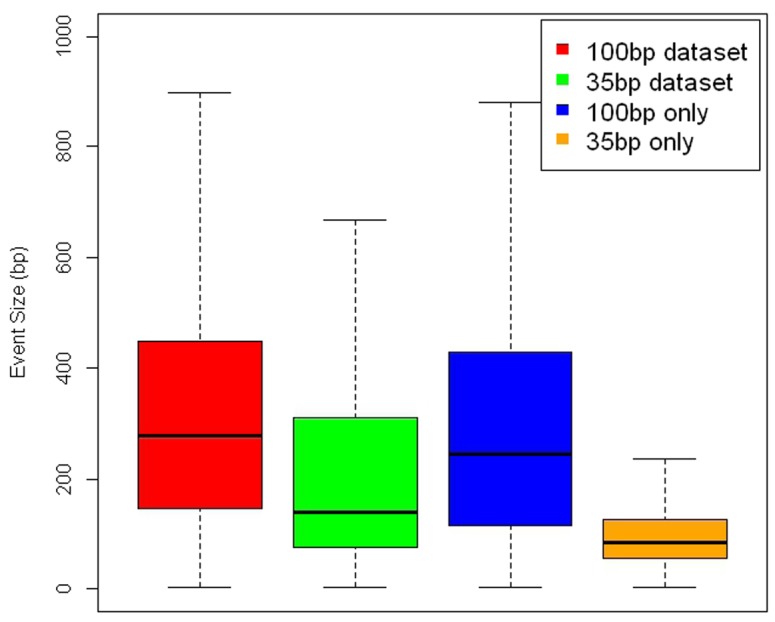
Size distributions of events detected using short and long read data. The distributions of events sizes detected by SVMiner based on short and long read datasets (Red: based on long reads but overlap with events based on short reads; green: based on short reads but overlap with events based on long reads; blue: events detected only from long reads; orange: events detected only from short reads).

With further development in technology, the length of sequence reads has increased over the years for many platforms. We therefore evaluate how experimental parameters such as read length and library insert size may affect the discovery of structural variants. This sample (NA18507) we have analyzed earlier has been sequenced several times using Illumina platforms. In our experiments, we considered two such datasets. In addition to data set I, which consists of paired-end short reads of 36∼41 bps and an average insert size of 200 bps (SRA000271), we tested SVMiner on the second dataset that consists of paired-end reads of 100 bps and an insert size of 500 bps (SRX016231). From this long read data (dataset II), SVMiner identified 6236 deletion events with 2550 homozygous deletions and 3686 heterozygous deletions. The overall overlap rate between results from the two datasets is around 44% (Figure 3, bottom panel, left). Intuitively, the overlap rate is not that high, which may be due to the differences in the datasets using different platforms, or due to the limitations of our algorithm. We further tested another program BreakDancerMax on both datasets. It turns out that the overlap rate of BreakDancerMax (34%–37%) is even lower (Figure 3, bottom panel, right). In fact, the level of consistency between the two approaches on the same datasets is much higher than the level of consistency of either algorithm on the two different datasets. For example, using the dataset with short reads, the fraction of overlapped events predicted by the two approaches is around 84%. After visual inspections of some predicted regions using IGV [26], we concluded that the two datasets have somewhat different mapping results for discordant reads. For example, Figures S5 and S6 provide examples of events detectable by one type of data but not by the other. Also, we found several instances where mapping qualities of reads in a possible deletion area are all zero in one dataset, while the other dataset has plenty of high quality discordant reads. One possible explanation is that in addition to the large deletions, there are possible small indels, which affect the mapping quality score differently for reads with different lengths. Another possibility is that smaller deletions may not be detectable using longer reads with a large insert size. Indeed, when examining event size distributions, many small events were only detected by shorter read data (Figure 4). This is because for longer insert datasets, it is more difficult to detect smaller deletions due to fluctuations in read pair insert lengths. Overall, our results illustrate that the capability of any computational method in detecting deletion events is constrained by technological platforms and mapping results. For paired-end sequence data, read length and insert size may affect the final set of detectable structural variants. To see the individual effect by decoupling read length and insert size, we simulated three libraries with the only differences are insert sizes (200, 500, 5 k) and tested SVMiner on these datasets with the same set of 190 embedded deletion events. Results show that small to medium sized deletions may not be detected using data with large insert size (Table S1). This is mainly because for small events, both features will be affected by the large insert size. Other features such as number of singletons may provide more robust signals in this case.

### Comparison with other approaches

Many approaches have been developed for calling structure variants. In addition to MoDIL, we chose to compare our results mainly with BreakDancerMax(BDM) and VariationHunterWeighted (VHW) on specimen NA18507, which has been studied intensively recently by a few groups [14], [17], [20], [23]. The three variants of VariationHunter are not independent. Only VHW was used to construct reliable dataset R_II later. Therefore, only results from VHW were considered in this subsection. We compared our results to theirs, either by running their methods on the same datasets, or by comparing their published results to ours. Because no ground truth is available for this dataset, it is difficult to directly compare results from different algorithms. We constructed three different sets of highly reliable deletion events, and compared the three approaches by treating these reliable set as ground truth. First, the 1000 genome project (1KG) has recently released a set of high quality population level structural variant polymorphisms based on results predicted by many different algorithms [3], [24]. If any algorithm tested here detects a deletion from the sample we have that is overlapped with an event in the 1KG set, it is very likely that the event is a true event. Therefore, we first calculated the intersection of the 1KG set with the predicted events from each of the three methods, and then took the union of these three intersections as the true events (denoted as R_I), with the understanding that the final set is most likely a proper subset of the true events from this sample. The second set (R_II) consists of all the events detected by at least two of the three algorithms, based on the belief that if an event is detected by at least two algorithms, it is more likely to be a true event. The third set (R_III) consists of the validated events from Kidd et al. [23]. However, one should notice that Kidd et al. used a very different technology (large fosmid clones), and it is only effective for large events. Therefore, the total number of deletions in R_III is only 155. Given the constructed sets of reliable events, we assessed the precisions (correct predictions out of all predictions) and recalls (correct predictions out of all true events) of each method, and compared their F_scores (the harmonic mean of precision and recall). Because neither BreakDancerMax nor VariationHunterWeighted could separate heterozygous deletions from homozygous deletions, we combined these two groups together and investigated the overall prediction performance, which is given in Table 4.

**Table 4:** Comparison of deletion results from three different approaches.

Constructed set (#)	method	# predictions	# true positives	Precision	recall	F-score
R_I (3412)	SVM	6279	2947	0.469	0.864	0.608
	BDM	6354	2614	0.411	0.766	0.535
	VHW	8897	3250	0.365	0.953	0.528
R_II (5504)	SVM	6279	5114	0.814	0.929	0.868
	BDM	6354	3873	0.610	0.704	0.653
	VHW	8897	5141	0.578	0.934	0.714
R_III (155)	SVM	6279	11	0.002	0.071	0.003
	BDM	6354	11	0.002	0.071	0.003
	VHW	8897	13	0.001	0.084	0.003

The three constructed sets of reliable events are: R_I) intersection of events from the 1KG project and events predicted by any method; R_II) events predicted by at least two methods; R_III) events from Kidd et al. The three approaches are SVMiner (SVM), BreakDancerMax (BDM) and VariationHunterWeighted (VHW).

The total numbers of deletion events reported by the three methods vary noticeably, ranging from ∼6200 to ∼8900. This may be due to the fact that in addition to differences in different algorithms per se, other factors such as differences in mapping tools, limitations on the maximum sizes of events, may also contribute to the discrepancy. Based on R_I and R_II, SVMiner significantly outperformed both BreakDancerMax and VariationHunterWeighted with the highest F_scores (SVMiner: 0.61 & 0.87, BreakDancerMax: 0.54 & 0.65, VariationHunterWeighted 0.53 & 0.71, more details can be found in Table 4). SVMiner and BreakDancerMax had similar numbers of predictions, but BreakDancerMax had a lower precision and a lower recall on both R_I and R_II. VariationHunterWeighted predicted many more events, therefore, had better recalls. But it suffered from low precisions. Because the total number of events in R_III is very small, the number of overlaps from any approach is small (11 to 13). R_III cannot be used in distinguishing the three approaches.

We also compared these three methods on a simulated data with known ground truth. As mentioned earlier in comparing SVMiner and MoDIL, SVMiner predicted a total of 153 deletions, all of which are from the 190 embedded deletion events, yielding a precision of 100% and a recall of 80.5%. BreakDancerMax detected 147 events and all of them are embedded events. VariationHunter predicted 185 events, of which 177 were embedded events. Overall, for the simulated data, SVMiner is slightly better than BreakDancerMax, while VariationHunter reports some false positives, but with gains in recalls in this case.

### Inversion events

**Figure 5:**
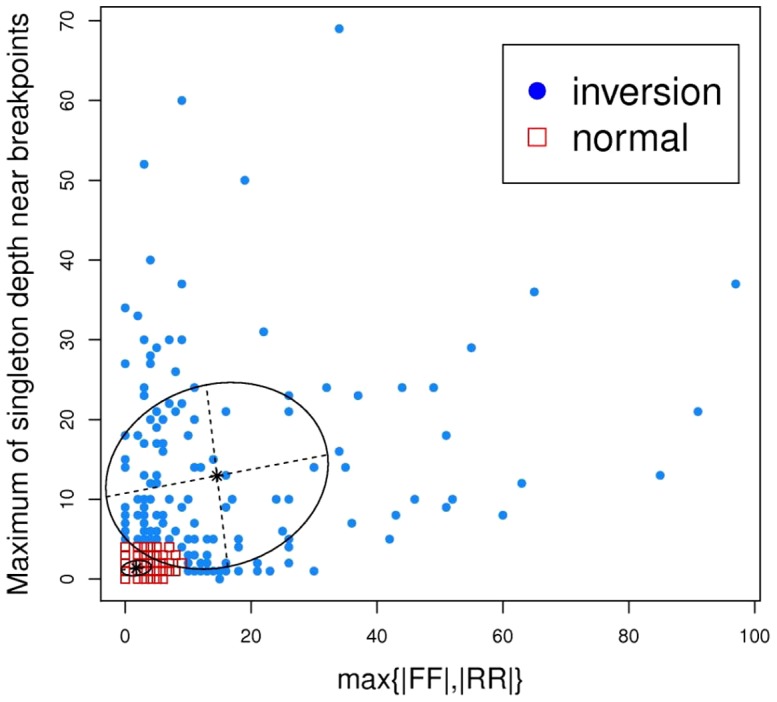
Inversion results. The result of the predicted inversions for the NA18507 specimen. The blue points are predicted inversions and the red points are classified as no events.

To test the capability of SVMiner in detecting inversions, we run the program on two datasets: dataset I and a simulated dataset. For dataset I, the total number of predicted inversion events is much smaller than the number of deletions. SVMiner initially identified 263 candidates, of which 148 were predicted as inversions after the clustering phase of our algorithm. Since the original paper by Bentley *et al.* did not report inversion results and coordinates of predicted inversions were not available for the BreakDancer algorithm, we could not compare our findings with theirs. The numbers of inversions predicted by three variations of VariationHunter vary greatly (from 181 to 504). The overlaps of our predicted events with those by VariationHunter are low (Table 5), which reflects that inversions are much harder to capture. The three variants of VariationHunter have large number of overlaps, because these three algorithms are not independent and share a common component. Neither SVMiner nor VariationHunter has detected many events reported by Kidd *et al.* We further investigated the impact on sensitivity caused by 1) the mapping ambiguity, 2) the filtering step described in the Materials and Methods section and 3) the final classification step. None of the steps significantly affected the number of overlaps, which suggests that the discrepancy between results by SVMiner (as well as VariationHunter) and results from Kidd et al. primarily is due to the differences in technologies used by the studies. Capability of computational approaches is limited by technologies that generate data. Figure 5 shows classification results by SVMiner, which illustrate that events with high singleton coverage and/or large numbers of discordant pairs will likely be classified as inversions and events that cluster near the origin tend to be labelled as normal events (i.e., not inversions). The results are in line with expectations because true events should have high level of singleton coverage and/or large numbers of discordant pairs while regions of no variation will likely not have inverted discordant pairs or accumulations of singleton reads. To further evaluate the performance of the proposed algorithm, we compared the results of three algorithms (SVMiner, BreakerDancerMax and VariationHunterWeighted) on a simulated dataset. SVMiner predicted a total of 189 inversions, and 170 of them overlapped with the 202 embedded events (precision: 89.9%, recall: 84.2%, F_score = 87.0%). BreakerDancerMax predicted 214 events and 148 of them overlapped with embedded inversions (precision: 69.2%, recall: 73.3%, F_score = 71.2%). VariationHunterWeighted only predicted 41 inversions of which 34 overlapped with the true events (precision: 82.9%, recall: 16.8%, F_score = 28.0%). We had made every effort to run VariationHunter correctly and used the same configuration as the one when analyzing real data, but it is unclear why the recall was so low. Overall, SVMiner performed the best on this dataset.

**Table 5:** The overlaps among predicted inversion events.

Kidd (82)	VHW (504)	VHU (433)	VHP (181)	
1	12	12	6	SVMiner (148)
	10	5	1	Kidd (82)
		351	151	VHW (504)
			141	VHU (433)

The overlaps among predicted inversion events by SVMiner, three versions of VariationHunter, and the events detected using a different technology (Kidd et al). The total number of events by each approach is provided in the parentheses.

## Discussion

Our results show that SVMiner is well suited for detecting both homozygous and heterozygous deletion events. It is more difficult to assess the efficacy of inversion detection, but SVMiner's detection of the inversion events in the simulated dataset lends credence to the choice of feature values for detecting inversions within the clustering framework. Of the methods we compared to SVMiner, only MoDIL had the ability to detect heterozygous deletions. Furthermore, not only did SVMiner clearly outperform MoDIL on both the chromosome X dataset and the simulated dataset, but the time needed for the analysis was much shorter for SVMiner. Since MoDIL is not suited for detecting large deletions, it is possible that its performance suffered because of this limitation.

Regarding the performance of SVMiner on NA18507, the results suggest that one can have high confidence in SVMiner's predictions. Compared to BreakDancer and VariationHunter, SVMiner had the highest F_scores when comparing with events from the 1000 genome project or events detected by at least two approaches. Since SVMiner utilizes a model-based clustering framework, it bases its predictions on patterns exhibited by the input dataset, thus freeing the user from any need to manually adjust parameters based on cluster characteristics. Variant cluster signatures may differ across different datasets, and SVMiner is well suited to take advantage of such variation. Although in this study, we did not filter the predictions using the membership probabilities, we have demonstrated that events with more accentuated features in general have lower uncertainties. In practice, depending on their need, researchers can use the probabilities to select a smaller set, but more reliable events for further investigations.

For the comparison of long and short read datasets with different insert lengths, we found that around 40% of events detected by SVMiner using the two datasets were in common. The overlap of deletion events detected by BreakDancer from these two datasets was even lower (∼30%). This suggests that although both programs are capable of detecting deletions using different read lengths and insert sizes, the set of events that can potentially be detected highly rely on the input datasets. The comparisons with events from Kidd et al further demonstrated limitations of different technologies. Unlike SNP calling platforms and algorithms which normally can achieve very high accuracy, further development in technologies and improvement in algorithms are greatly needed for structural variation calling. A more complete catalogue of common structural variants with more accurate breakpoint information in different populations will also greatly help the heterozygosity predictions.

Future work will investigate other features to be included in the model and features for the refinement of breakpoints of SVs at the nucleotide level. For example, the number of singletons can also be used in predicting deletions, which may provide more evidence for real events. On the other hand, adding more features will increase the complexity of the model. The trade-off needs further investigations. One of the contributions of our approach is that it can genotype simple structural variation events (*i.e.*, deletions), so we will also investigate ways to predict the heterozygosity of events other than deletions. Very recently, similar attempts are being conducted by other researchers. For example, when we were preparing the manuscript, Hormozdiari *et al.* [18] extended their VariationHunter algorithm to identify transposon events [27], in which they also took into account the diploid structure of the human genome.

It is known that other factors, such as local G+C content and local repetitive structures [14], can greatly affect local read mapability. Therefore, even given a specific type of structural variation (*e.g.*, heterozygous deletion), the expected value of a particular feature (*e.g.*, normal read depth) may vary, which in turn may affect our final predictions. We will investigate approaches to account for these factors. Furthermore, the mixture model in our approach assumes normal distributions of all features, which may not hold in practice. The effect of this violation of normal assumption on the final predictions was not thoroughly investigated in this study. By considering the relationships of the expected value of read depths and local sequence characteristics (e.g., G+C conent), it is possible that we can derive or transform the raw values of our features to better fit the normal assumption. Lastly, we will extend our approach to detect structural variants not described in this paper, such as tandem duplications [28] and translocations [6].

## Conclusions

We have presented a framework called SVMiner for detecting genomic structural variation using model based clustering. In addition to separation distances and read pairs' orientations, SVMiner automatically defines and collects additional features for different types of structural variation candidates, and utilizes these features in a probabilistic model to cluster and classify each candidate variant. We applied the approach on three real datasets generated using next generation sequencing technologies and results demonstrated that the cluster features we defined for each type of SV events were appropriate for identifying SVs. In comparison with previous studies, SVMiner has achieved much higher F_scores than existing approaches in detecting and genotyping deletions. Results on inversion detections suggest that further improvements or new developments for other structural variant events are needed.

## Materials and Methods

### Candidate generation for deletions

After being mapped, pairs with high mapping quality (score≥30) and with a separation distance greater than the insert size plus *x* × standard deviation are regarded as discordant pairs for candidate deletion events, where the insert size is approximated using the mean of the mapped distances and *x* = 3 or 4, which is consistent with the analyses in the original papers. To generate candidate SV events, overlapped discordant deletion pairs are clustered based on their lengths and positions. More specifically, we require that overlapping discordant pairs must have similar mapped distance lengths. This is necessary because discordant pairs from the same deletion event are more likely to have similar sizes. In addition, they should also have similar positions. Therefore, we require that every read pair in one cluster must overlap with all other read pairs within the same cluster. This requirement removes read pairs from a cluster that could, for example, overlap with one pair, but not with others. This aberrant read pair may have been mapped by chance. Furthermore, only pairs with a mapping distance less than 100 kilobases are considered in this study. SVMiner can, however, detect deletions larger than 100 kb by adjusting this maximum allowed pair size. Finally, only clusters with at least three discordant pairs are considered as candidate deletion events. We refer to this step as the “filtering” step.

### Feature collection for deletions

For each candidate deletion event, we define and collect two features for classification: 1) the number of discordant pairs of the candidate cluster and 2) the average depth of concordant pair reads within the cluster region, based on the following observations. If the event is indeed a homozygous deletion (*i.e.*, regions where both chromosomes/haplotypes deleted), one would expect that the number of discordant pairs should be close to the expected coverage of the region. At the same time, within the deleted region, the depth of reads should be very close to 0 if not 0. On the other hand, if the event is a heterozygous deletion, one would expect that the number of discordant pairs as well as the depth of concordant pairs will be close to half of the expected depth in the region. In an unaffected region, high depth of concordant pairs and low depth of discordant pairs is expected. Similar observations have been made by other researchers (e.g., [14]), but few approaches have automatically collected these features based on these observations to incorporate them into a formal model. Figure S7 shows the distributions of concordant pairs and discordant pairs around a homozygous deletion (Fig. S7A) and a heterozygous deletion (Fig. S7B) from a real dataset [14], which are consistent with the observation. The two features are then used by the model-based clustering algorithm, which will predict three possible outcomes for deletion events: 1) homozygous deletions, 2) heterozygous deletions, and 3) no events.

### Candidate generation for inversions

The candidate inversion events can be determined in a similar way to the aforementioned method of determining candidate deletions. First, only inverted pairs with the same orientation and with high mapping quality (score≥30) are retained. To form a cluster, in this study we require there to be at least three overlapping pairs discordant by orientation, which is a user specified parameter in the implementation of SVMiner. We further require that a candidate inversion has to satisfy the following conditions. For an inverted discordant pair with forward-forward (FF) orientation, it should theoretically have a greater mapped distance than other FF discordant pairs in the cluster with larger genomic coordinates (See Figure S8 for an illustration). This property should also hold for any reverse-reverse (RR) oriented pair. If a read pair *p* is the first FF pair in the cluster and *q* is the last pair according to the coordinates of their left mates, the distance between the start positions of the left mates of *p* and *q* should be less than the insert size of *p* (Figure S8). This relation should also hold for the right mates of FF reads and for the RR pairs. Also, if both FF and RR pairs exist in a cluster, the left mates of all FF pairs should be located before the left mates of all RR oriented pairs. Lastly, all inverted pairs with mapped distances greater than 500 kilobases (provided as parameter in the program) are discarded in this study. This step is referred to as the “filtering” step for inversion.

### Feature collection for inversions

For each candidate inversion, we define two intuitive features to be used by the model-based clustering method. The first feature is defined as follows:
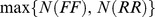
where *N*(FF) is the number of FF reads that form the cluster and *N*(RR) is the number of RR reads. Theoretically, there should be both FF and RR discordant pairs that should support an inversion event (Figure S9A), and the number of which should be close to the average read depth in the region. Occasionally, however, there may only be RR or FF read pairs in a cluster that support a true inversion. For example, Bentley *et al.* [14] provided an example of a validated inversion event that is only supported by FF pairs (Figure S9B). The lack of alternate discordant reads may be explained by lower read coverage in the region where the other types of pairs are expected, or they may be caused by the deletion from the reference of nearby repeated regions.

The second feature we define is related to the coverage of singleton reads near the cluster, which are reads whose mate pair does not map to the reference sequence (for shorter reads), or whose mate pair has been split (for longer reads). For many SV events, including inversions, it is expected that the reads that cover the breakpoints in the donor genome will either 1) not map to the reference, or 2) align disjoint segments of a read to distinct locations on the reference genome and create a split read. Therefore, the mates of the unmapped/split reads, termed as singleton reads here, will be present near inversion boundaries, which was also described by others (e.g., [14]). Figure S9 shows the tendency of singleton reads to accumulate at inversion breakpoint boundaries.

We define this feature by measuring the number of singleton reads that cover areas near the expected breakpoints. For each FF pair, we define four windows, and for each base pair within each window, we measure the number of singleton reads with quality score≥30 that cover it. The first window is defined as the interval [*StartPosition*(Left) – α, *StartPosition*(Left)], where “Left” is the left read and α is a window size. For this window, we only consider the singletons that mapped to the + strand, and we use α = 300 for dataset I to capture an interval that is slightly larger than the expected insert size. The second window is defined as [*StartPosition*(Left), *StartPosition*(Left)+α] and for this window, we consider both (+,−) singletons because we suspect the left breakpoint of the inversion would be located near this window, and there could be (+) or (–) singletons whose mates do not map to the breakpoint. The third window is defined as [*StartPosition*(Right) - α, *StartPosition*(Right)], where “Right” is the right read. The fourth window is defined as [*StartPosition*(Right), *StartPosition*(Right)+1.5*α]. Because possible inversion breakpoints can be located within the fourth window, only forward (+) singleton reads of third window are informative while both (+) and (−) singleton reads from the fourth windows are informative. We extend the last window to increase the chances of including the right inversion breakpoint, since the range of a FF pair may not flank this breakpoint. If both FF and RR discordant pairs are present in the cluster, it is easier to find both breakpoints. However, the lack of one type of inverted pair hinders our ability to find both breakpoints, hence the larger size for the fourth window. For all RR pairs in the cluster, we define our four windows in a similar fashion. But for these pairs, the breakpoints can only be located within the first or third windows. Having defined a total of four windows for each discordant pair, we defined our second feature by taking the maximum, over all windows and all read pairs, of the number of singletons that cover all base pairs in each window. This feature is defined symbolically as
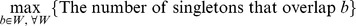
where *W* is a window defined for some paired read and *b* is a base pair that is located within *W*. 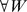 quantifies the set of all windows over all paired reads in the cluster.

The two features are collected automatically for each candidate cluster and then used by the model based clustering algorithm. Like deletion events, theoretically, inversions can also occur on either haplotype. However, the high variability in discordant pair coverage obfuscates genotyping of inversions (e.g., not every inversion event has FF and RR reads). Therefore, only two possible outcomes are considered for candidate inversions: predicted true inversions and normal regions.

### Model-based clustering algorithm

We assume that observed data points are generated from different component distributions. More specifically, each candidate event *x_i_* is a vector of two dimensions in our application, representing the values of two features in each type of event. Let 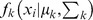 denote the probability density of the observation *x_i_* being from mixture component *k*, which is usually assumed to be a normal distribution with parameter mean *μ_k_*, and covariance *∑_k_*,
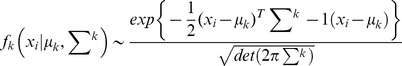
(1)For a vector of observations **x**, its log likelihood function is defined as:
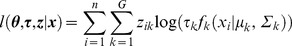
(2)where 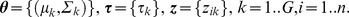 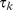 is the mixture probability of component *k*, 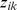 is an (unobserved) indictor variable that is 1 if 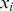 belongs to component *k*, *G* is the number of components.

The maximum likelihood parameters for Equation (2) are computed via the expectation-maximization (EM) algorithm (Dempster *et al.*, 1977). In the E-step, the expected values for *z_ik_* are calculated as:
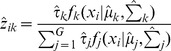
(3)where 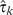, is the component probability estimated from the M step, and 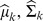 are the mean and covariance matrix for component *k* learned from the M step. These learned parameters are updated on successive iterations of the E and M steps. For classifying an observation, the component that maximizes the converged membership probability 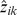 is the label assigned to the observation after the iterations terminate.

The distribution of each component can be further refined by specifying a smaller number of free variables in the covariance matrix Σ*_k_*, which is equivalent to specifying the geometric characteristics of each component. Evaluation of different models can be quantified by calculating the Bayesian Information Criterion (BIC) [29] value for each model, given the current dataset. One can refer to Fraley and Raftery [25] for a detailed discussion of different models. In our implementation, we have used the model without restrictions. In addition, we have tried a few different initialization methods (*e.g.*, assign class labels randomly or using different thresholds), and no much differences have been observed from different initializations. The threshold for convergence is 10^−6^.

### Generating simulated data

Using the chromosome 9 of the human reference genome, we generated two homologous chromosomes (*i.e.*, diploid) and randomly embedded 190 non-overlapping deletions and 202 inversions of varying lengths (Table S2). Of the 190 embedded deletions, 94 were homozygous and 96 were heterozygous. The two chromosomes were then sheared into reads of length 36. The average insert length between a pair of reads is 200 with the standard deviation of 20, which closely mimics the real data at hand. Point mutation rate is 0.1% and sequencing error rate is 0.5%. After alignments, the mean read depth is around 31×.

## Supporting Information

Table S1 **Insert size analysis.** We assessed the capability of SVMiner in detecting deletions using sequence libraries of different insert sizes. We created 3 simulated libraries (200, 500, 5 k), in a similar manner to the data generation steps described in the Methods and Materials section. To declare discordant pairs, we have used *x* = 4 times standard deviation (4sd) and 3 times standard deviation (3sd). Results show that small to medium sized deletions may not be detectable by discordant pairs with a large insert size.
(DOCX)

Table S2 **Events generated by the simulation study.**
(DOCX)

Figure S1 **Comparison of the mapping quality scores of read pairs from the two programs MAQ and BWA based on randomly selected two million read pairs from dataset I.** The correlation coefficient between quality scores of the two programs is 0.78.
(TIF)

Figure S2 **The distributions of the two features for the three types of events (top: predicted normal events; middle: predicted heterozygous deletions; bottom: predicted homozygous deletions).** Events predicted as being normal generally have low discordant pair support and relatively high concordant pair depth. Heterozygous regions typically have significant support from both concordant and discordant pairs. Homozygous deletions are expected to have high discordant pair support and very low concordant pair support. The results in general agree with expectations. At the same time, it is apparent there are also normal events with low concordant pairs, heterozygous deletions with low concordant pairs and low discordant pairs, homozygous deletions with low discordant pairs. Those are data points that near the origin and/or class boundaries, which normally have high uncertainty values associated with their classification as shown in Figure 2B.
(TIF)

Figure S3 **Membership probability distributions of predicted normal events with average concordant depth less than 6 (Left) and greater or equal to 6 (Right).** The two distributions are significantly different (2-sample K-S test, p<10^−10^). On the left, the membership probabilities are generally less than 0.90; on the right, the membership probabilities are generally greater than 0.90. When the data points have a low average concordant depth, there is greater uncertainty whether the low coverage is due to 1) an actual deletion or 2) some other sources (such as high G+C content). The membership probabilities in general capture the uncertainty well and can be utilized to select a subset of more reliable predictions.
(TIF)

Figure S4 **An event that was identified by SVMiner and MoDIL but with different genotype calling.** It was predicted as a homozygous deletion by SVMiner, but a heterozygous deletion by MoDIL. On the left (a), the mapped read pairs along the chromosomes. Blue indicates concordant pairs, and red represents discordant pairs. Dashed lines are breakpoints of SVMiner and dotted lines are breakpoints of MoDIL. On the right (b), we reconstructed the signals defined by MoDIL based on its breakpoint information, which are the distribution of separation distances of mapped read pairs within the breakpoints. We suspect that MoDIL calls this one as heterozygous deletion because the mapping distances clearly come from two distributions of similar sizes, corresponding to the two haplotypes (one with a deletion and the other with no deletion).
(TIF)

Figure S5 **Visualization of an event that was only detected based on longer read data.** Upper panel is the mapping results of shorter read data and lower panel is the results of longer read data. Discordant paired reads are in brown color.
(TIF)

Figure S6 **Visualization of an event that was only detected based on shorter read data. Upper panel is for shorter read data and lower panel is for longer read data.**
(TIF)

Figure S7 **Examples of deletion events.** (a) A homozygous deletion and (b) a heterozygous deletion on chromosome X of a NA07340. The abnormally spaced discordant read pairs are represented as red lines, while the concordant pairs are seen as blue lines. The deletion event in (b) has only occurred on one haplotype, thus explaining the presence of concordant pairs within the affected region.
(TIF)

Figure S8 **A synthetic example to illustrate additional requirements for candidate inversion clusters.** Red reads are FF reads and blue ones are RR reads. All read pairs are ordered according to the start positions of their left mates. The insert size of the first pair is expected to be greater than or equal to the insert size of the last pair in the FF group, i.e., *IS_1_*> = *IS_n_*. The maximum distance (D_1,n_) between left mates of the FF read group is expected to be less than or equal to the mean insert size. If both the FF read group and the RR read group exist, the starting positions of all left mates of the FF group should be in front of any left mate in the RR group (i.e., Pos1< = Pos2). Similarly, one can derive their relationships for right mates and for the RR read group.
(TIF)

Figure S9 **Examples of inversion events, one on chromosome 1 of NA18507 (a) and the other is on chromosome X of NA07340 (b).** The FF discordant pairs are red and the RR pairs are green. Singleton reads are shown in dark blue and light blue, corresponding to forward reads and reverse reads, respectively. Note that (b) lacks RR discordant pairs. Singleton reads tend to accumulate near the inversion breakpoints because their mates do not map to the reference sequence due to potential overlap with the breakpoints.
(TIF)
