# Supplementary material for: A Model-Based Clustering Method for Genomic Structural Variant Prediction and Genotyping Using Paired-End Sequencing Data
Source: PLoS One. 2012 Dec 27;7(12):e52881. doi: 10.1371/journal.pone.0052881 (PMC3531386; doi:10.1371/journal.pone.0052881)
Supplement: Table S1 — Insert size analysis. We assessed the capability of SVMiner in detecting deletions using sequence libraries of different insert sizes. We created 3 simulated libraries (200, 500, 5 k), in a similar manner to the data generation steps described in the Methods and Materials section. To declare discordant pairs, we have used x = 4 times standard deviation (4sd) and 3 times standard deviation (3sd). Results show that small to medium sized deletions may not be detectable by discordant pairs with a large insert size. (DOCX) [file pone.0052881.s001.docx]

| **Total # of true events** | **Dataset/parameter** | **# of predictions** | **Precision** | **Recall** |
| --- | --- | --- | --- | --- |
| 190 | (200bps, 4sd) | 153 | 1 | 0.805 |
|  | (200bps, 3sd) | 175 | 0.886 | 0.816 |
|  | (500bps, 4sd) | 178 | 0.888 | 0.832 |
|  | (500bps, 3sd) | 191 | 0.759 | 0.763 |
|  | (5kbps, 4sd) | 52 | 0.673 | 0.184 |
|  | (5kbps, 3sd) | 63 | 0.587 | 0.194 |
