# Supplementary material for: A Model-Based Clustering Method for Genomic Structural Variant Prediction and Genotyping Using Paired-End Sequencing Data
Source: PLoS One. 2012 Dec 27;7(12):e52881. doi: 10.1371/journal.pone.0052881 (PMC3531386; doi:10.1371/journal.pone.0052881)
Supplement: Table S2 — Events generated by the simulation study. (DOCX) [file pone.0052881.s002.docx]

|  | Deletions | | Inversions | |
| --- | --- | --- | --- | --- |
| Event size | homozygous | heterozygous | homozygous | Heterozygous |
| 100 | 8 | 9 | 10 | 9 |
| 250 | 9 | 9 | 8 | 8 |
| 500 | 7 | 8 | 8 | 8 |
| 750 | 9 | 8 | 8 | 10 |
| 1k | 8 | 9 | 8 | 8 |
| 2.5k | 10 | 9 | 9 | 9 |
| 5k | 9 | 8 | 10 | 8 |
| 7.5k | 8 | 9 | 9 | 10 |
| 10k | 8 | 8 | 9 | 8 |
| 25k | 8 | 9 | 9 | 9 |
| 50k | 6 | 6 | 7 | 6 |
| 75k | 4 | 4 | 4 | 4 |
| 100k | - | - | 3 | 3 |
| Total | 94 | 96 | 102 | 100 |
