# Supplementary figures and images for: A Model-Based Clustering Method for Genomic Structural Variant Prediction and Genotyping Using Paired-End Sequencing Data
Source: PLoS One. 2012 Dec 27;7(12):e52881. doi: 10.1371/journal.pone.0052881 (PMC3531386; doi:10.1371/journal.pone.0052881)

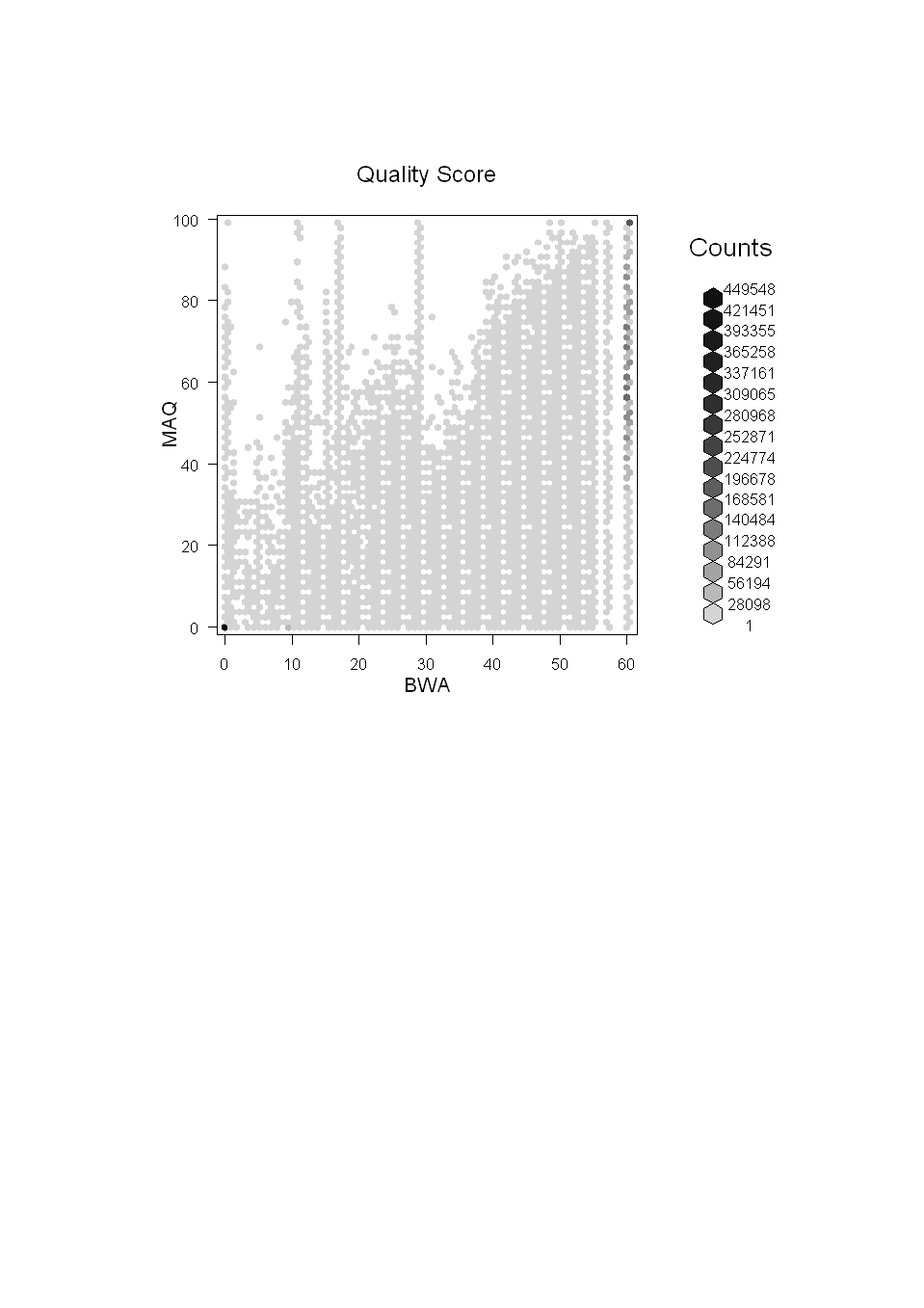

Supplement: Figure S1 — Comparison of the mapping quality scores of read pairs from the two programs MAQ and BWA based on randomly selected two million read pairs from dataset I. The correlation coefficient between quality scores of the two programs is 0.78. (TIF) [file pone.0052881.s003.tif]

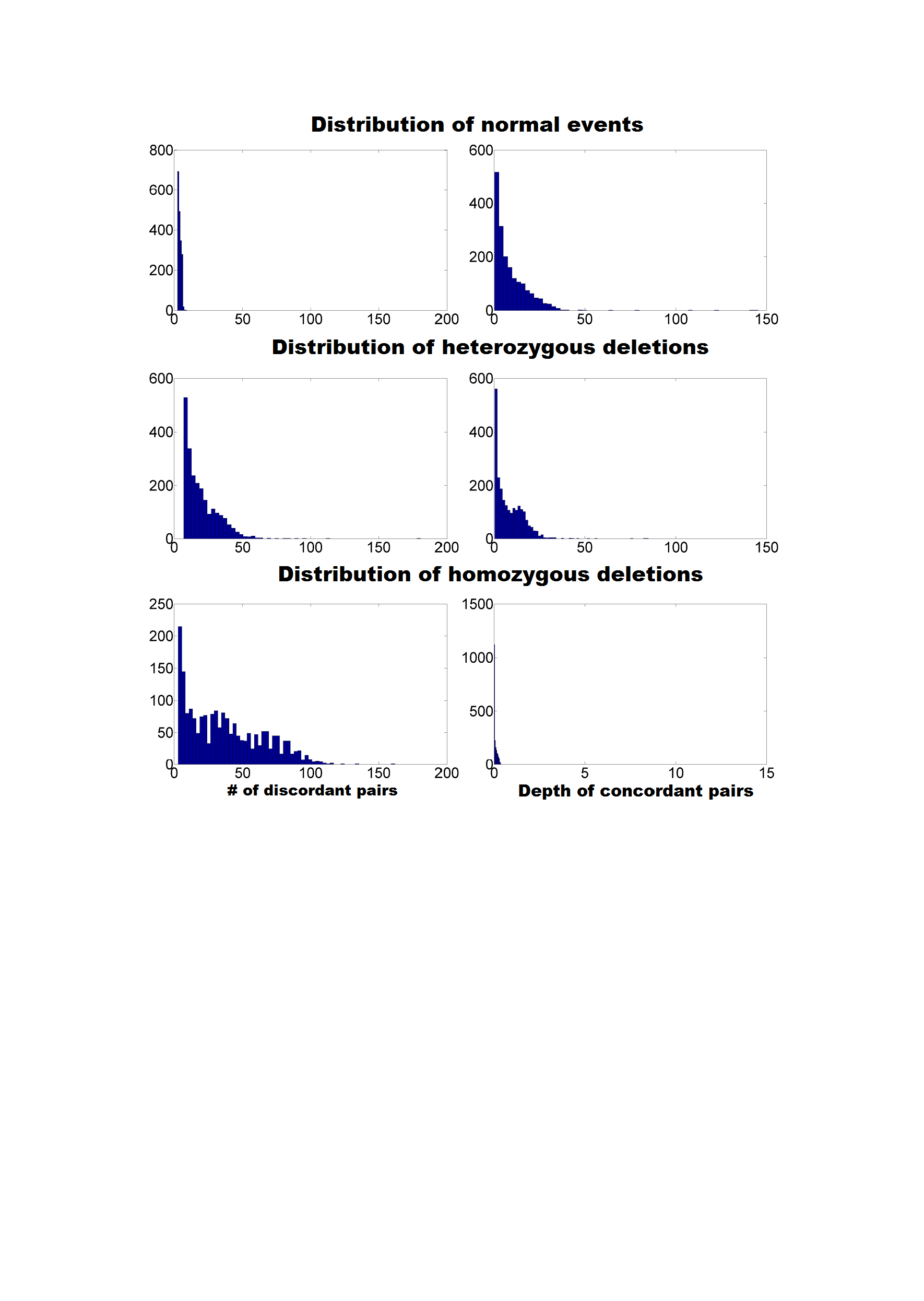

Supplement: Figure S2 — The distributions of the two features for the three types of events (top: predicted normal events; middle: predicted heterozygous deletions; bottom: predicted homozygous deletions). Events predicted as being normal generally have low discordant pair support and relatively high concordant pair depth. Heterozygous regions typically have significant support from both concordant and discordant pairs. Homozygous deletions are expected to have high discordant pair support and very low concordant pair support. The results in general agree with expectations. At the same time, it is apparent there are also normal events with low concordant pairs, heterozygous deletions with low concordant pairs and low discordant pairs, homozygous deletions with low discordant pairs. Those are data points that near the origin and/or class boundaries, which normally have high uncertainty values associated with their classification as shown in Figure 2B. (TIF) [file pone.0052881.s004.tif]

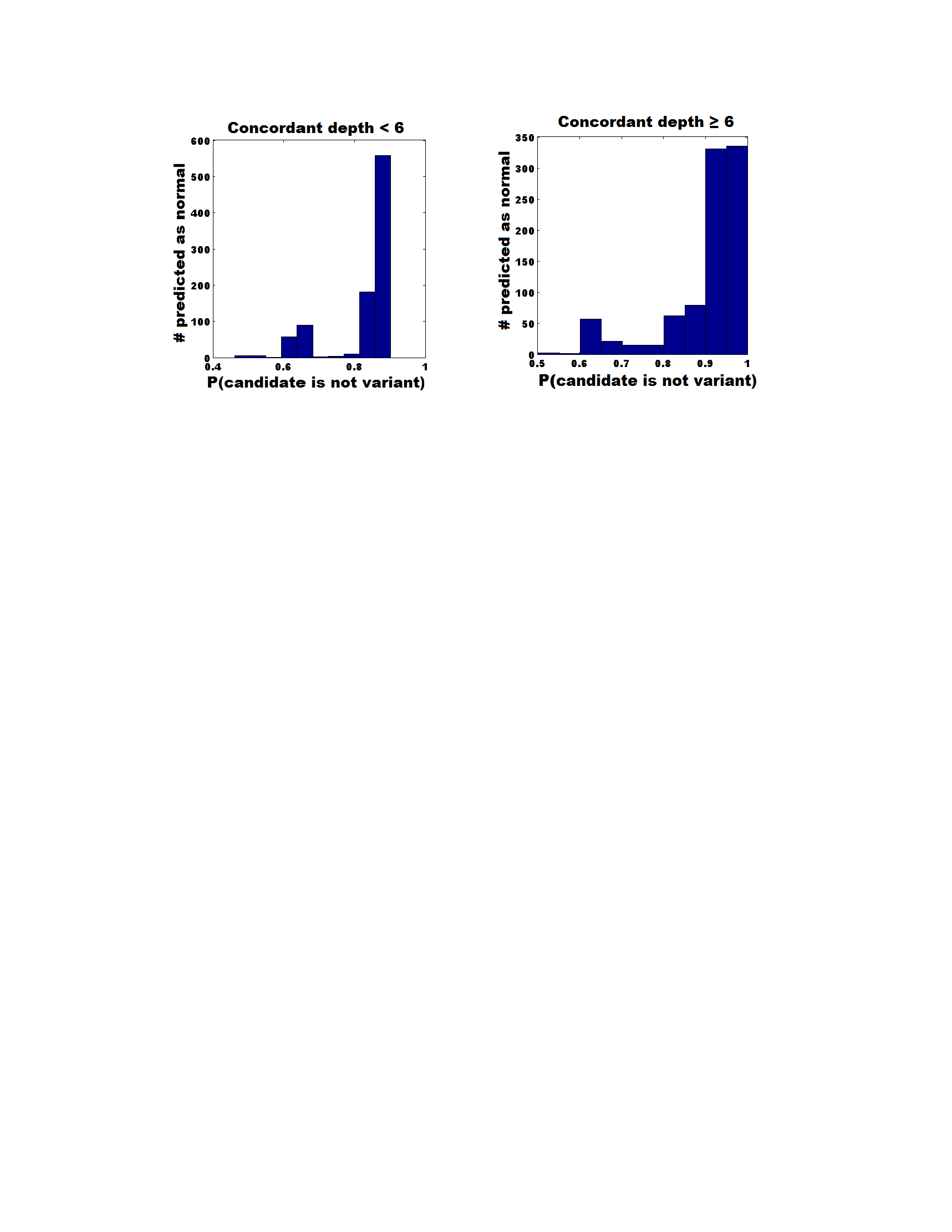

Supplement: Figure S3 — Membership probability distributions of predicted normal events with average concordant depth less than 6 (Left) and greater or equal to 6 (Right). The two distributions are significantly different (2-sample K-S test, p<10−10). On the left, the membership probabilities are generally less than 0.90; on the right, the membership probabilities are generally greater than 0.90. When the data points have a low average concordant depth, there is greater uncertainty whether the low coverage is due to 1) an actual deletion or 2) some other sources (such as high G+C content). The membership probabilities in general capture the uncertainty well and can be utilized to select a subset of more reliable predictions. (TIF) [file pone.0052881.s005.tif]

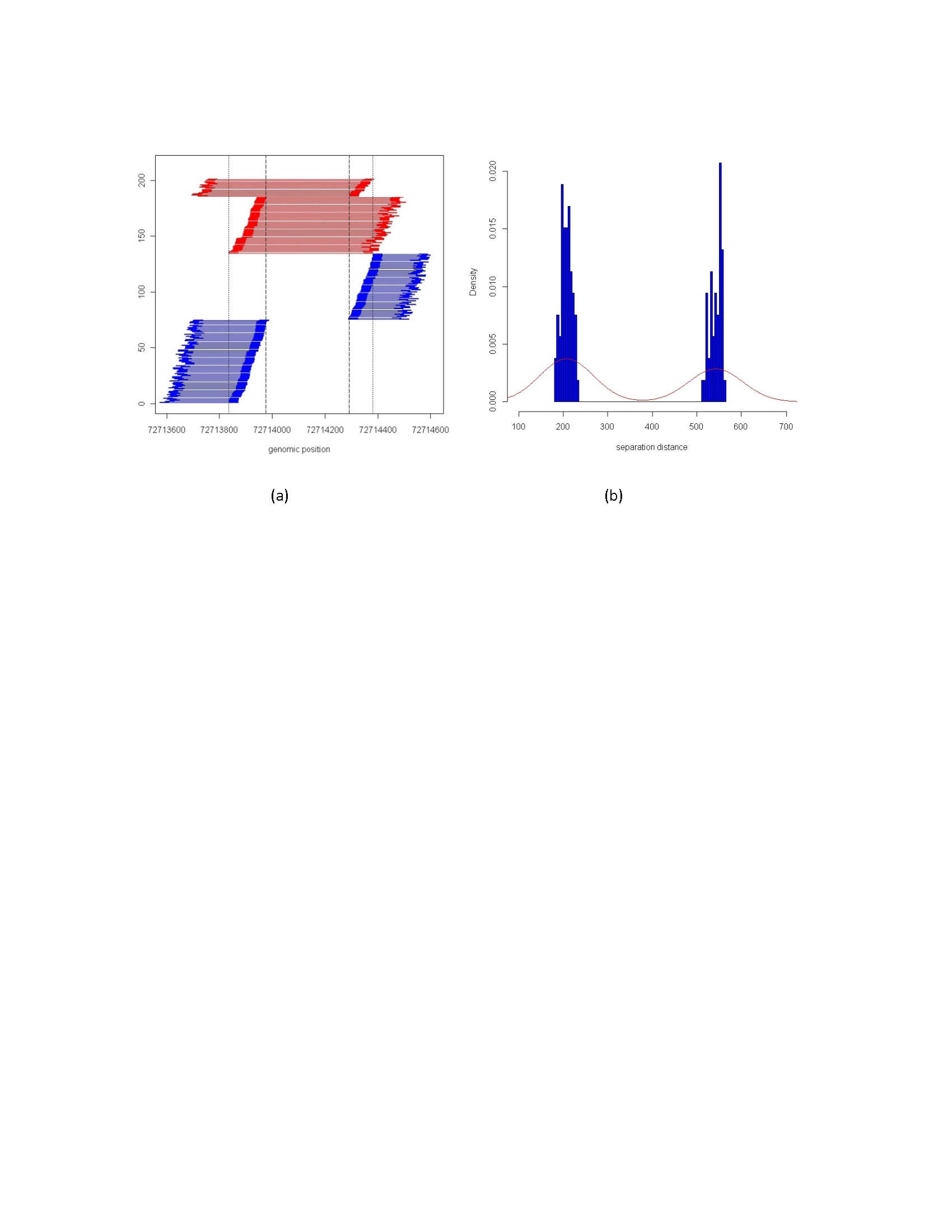

Supplement: Figure S4 — An event that was identified by SVMiner and MoDIL but with different genotype calling. It was predicted as a homozygous deletion by SVMiner, but a heterozygous deletion by MoDIL. On the left (a), the mapped read pairs along the chromosomes. Blue indicates concordant pairs, and red represents discordant pairs. Dashed lines are breakpoints of SVMiner and dotted lines are breakpoints of MoDIL. On the right (b), we reconstructed the signals defined by MoDIL based on its breakpoint information, which are the distribution of separation distances of mapped read pairs within the breakpoints. We suspect that MoDIL calls this one as heterozygous deletion because the mapping distances clearly come from two distributions of similar sizes, corresponding to the two haplotypes (one with a deletion and the other with no deletion). (TIF) [file pone.0052881.s006.tif]

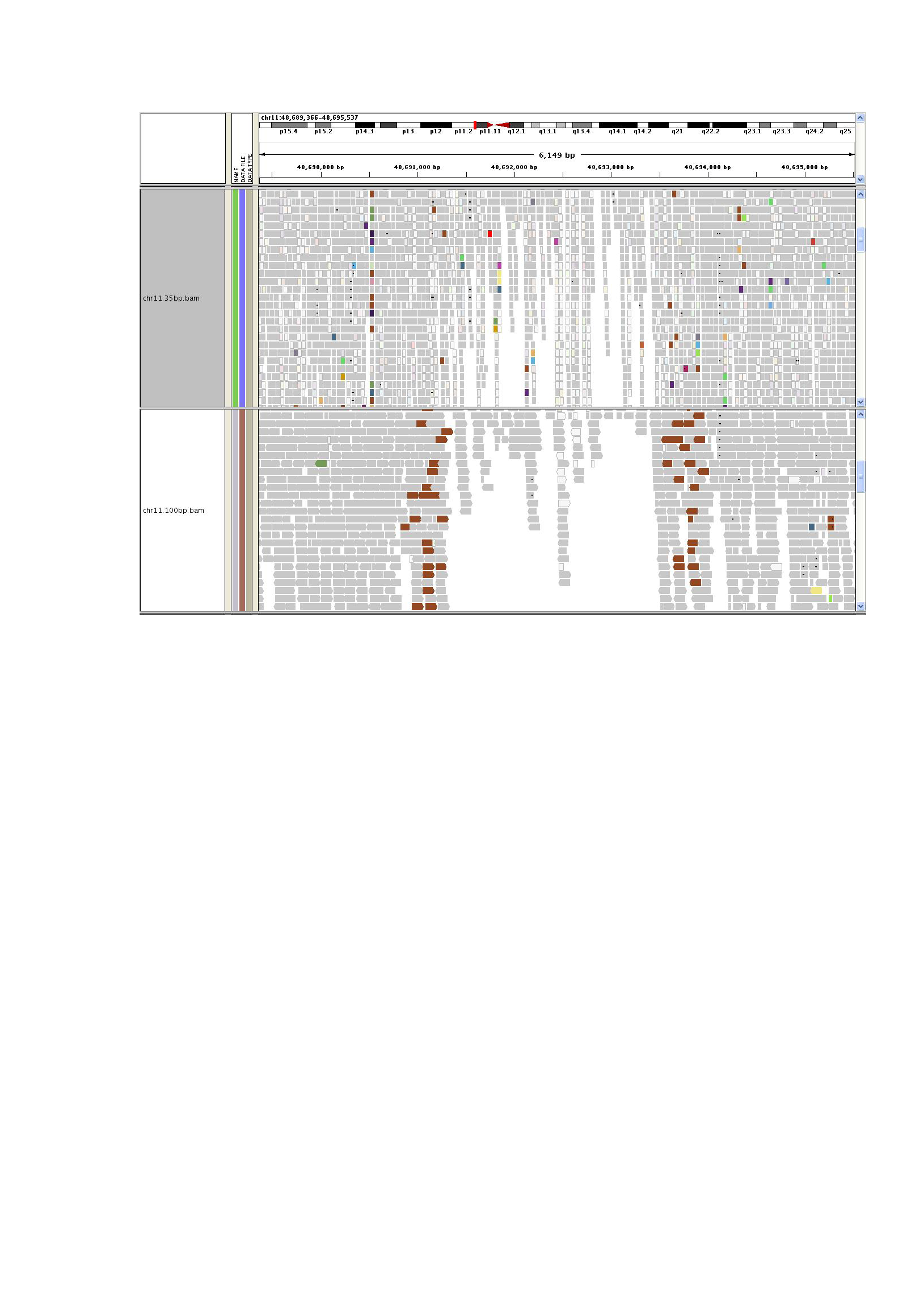

Supplement: Figure S5 — Visualization of an event that was only detected based on longer read data. Upper panel is the mapping results of shorter read data and lower panel is the results of longer read data. Discordant paired reads are in brown color. (TIF) [file pone.0052881.s007.tif]

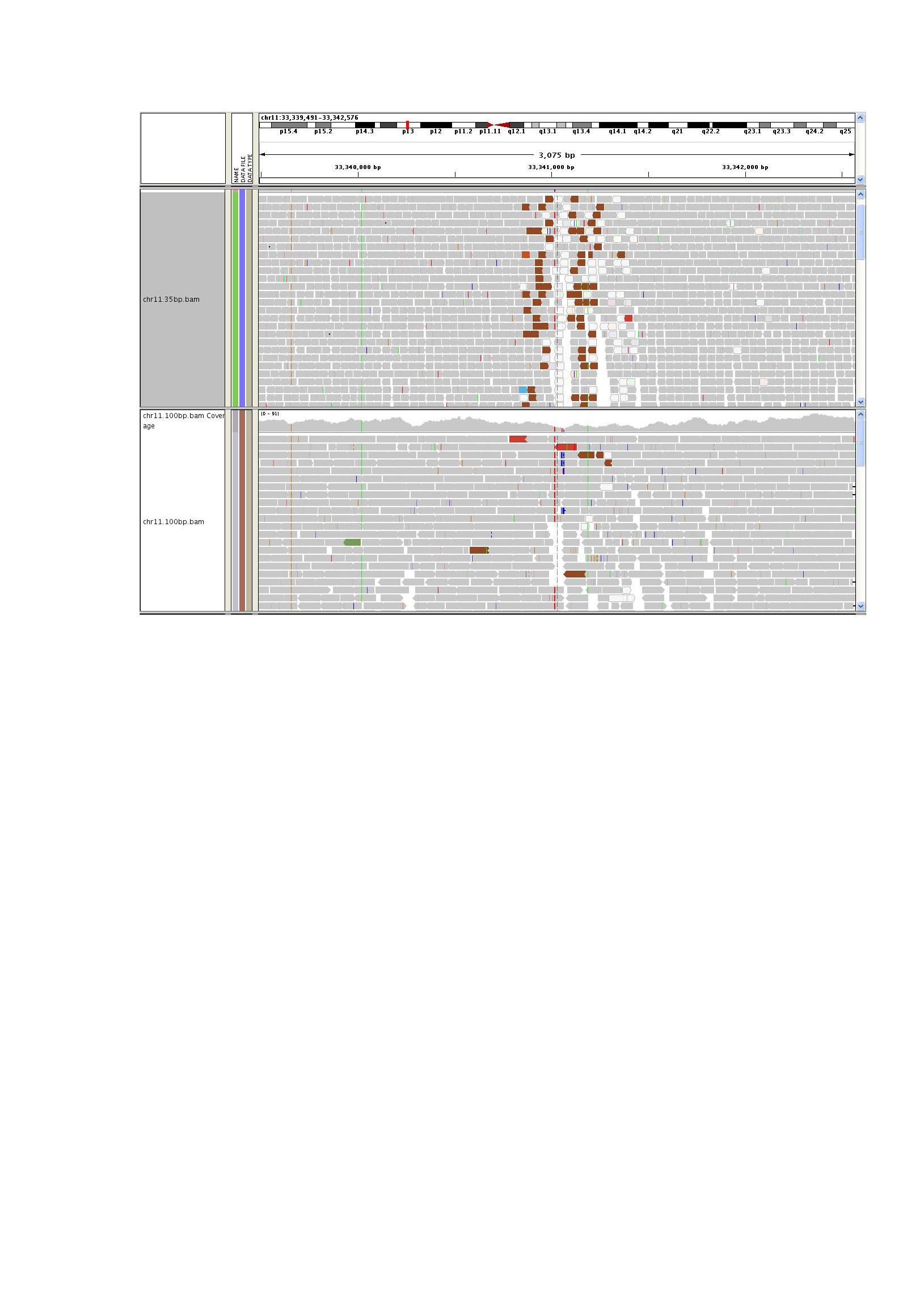

Supplement: Figure S6 — Visualization of an event that was only detected based on shorter read data. Upper panel is for shorter read data and lower panel is for longer read data. (TIF) [file pone.0052881.s008.tif]

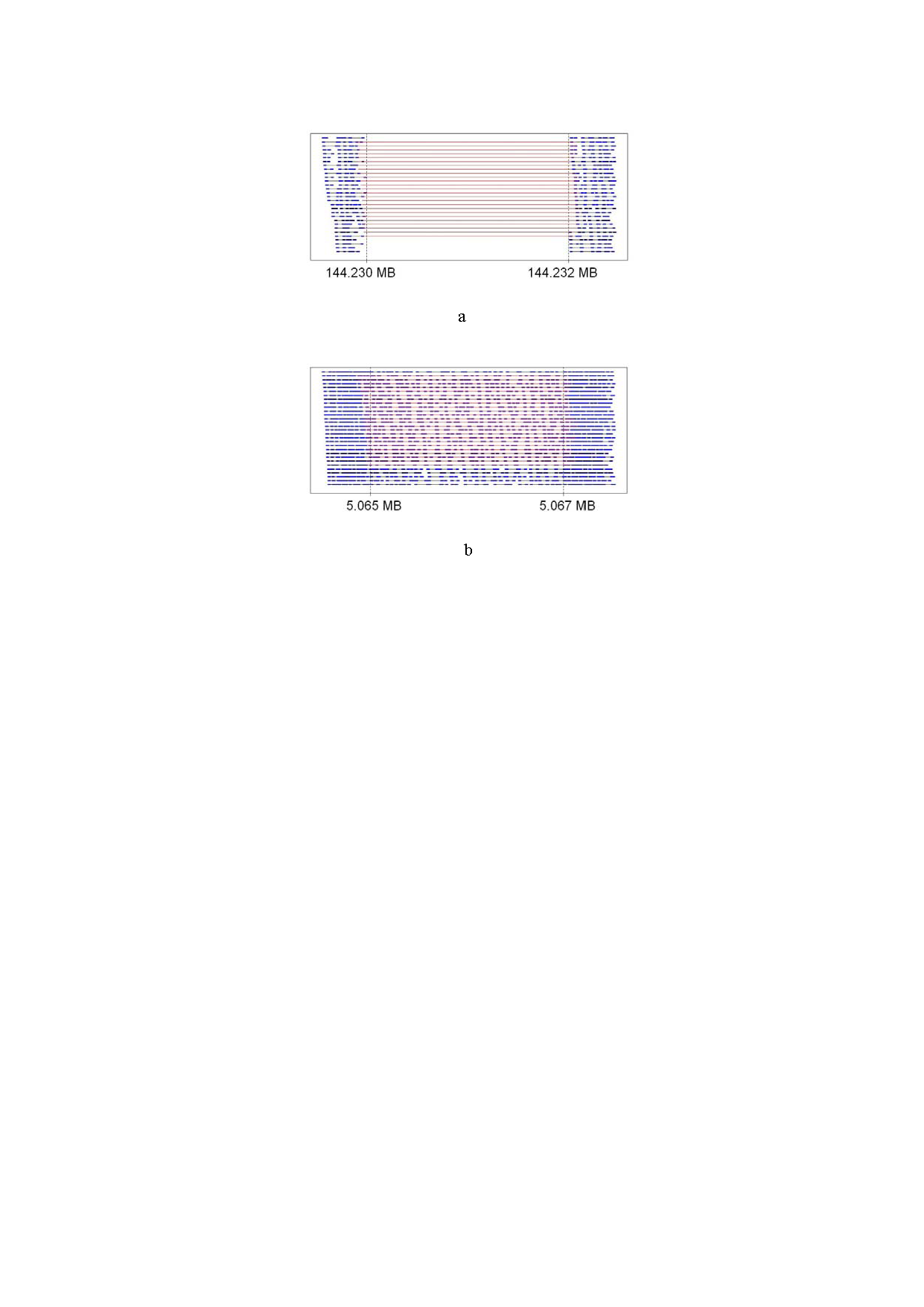

Supplement: Figure S7 — Examples of deletion events. (a) A homozygous deletion and (b) a heterozygous deletion on chromosome X of a NA07340. The abnormally spaced discordant read pairs are represented as red lines, while the concordant pairs are seen as blue lines. The deletion event in (b) has only occurred on one haplotype, thus explaining the presence of concordant pairs within the affected region. (TIF) [file pone.0052881.s009.tif]

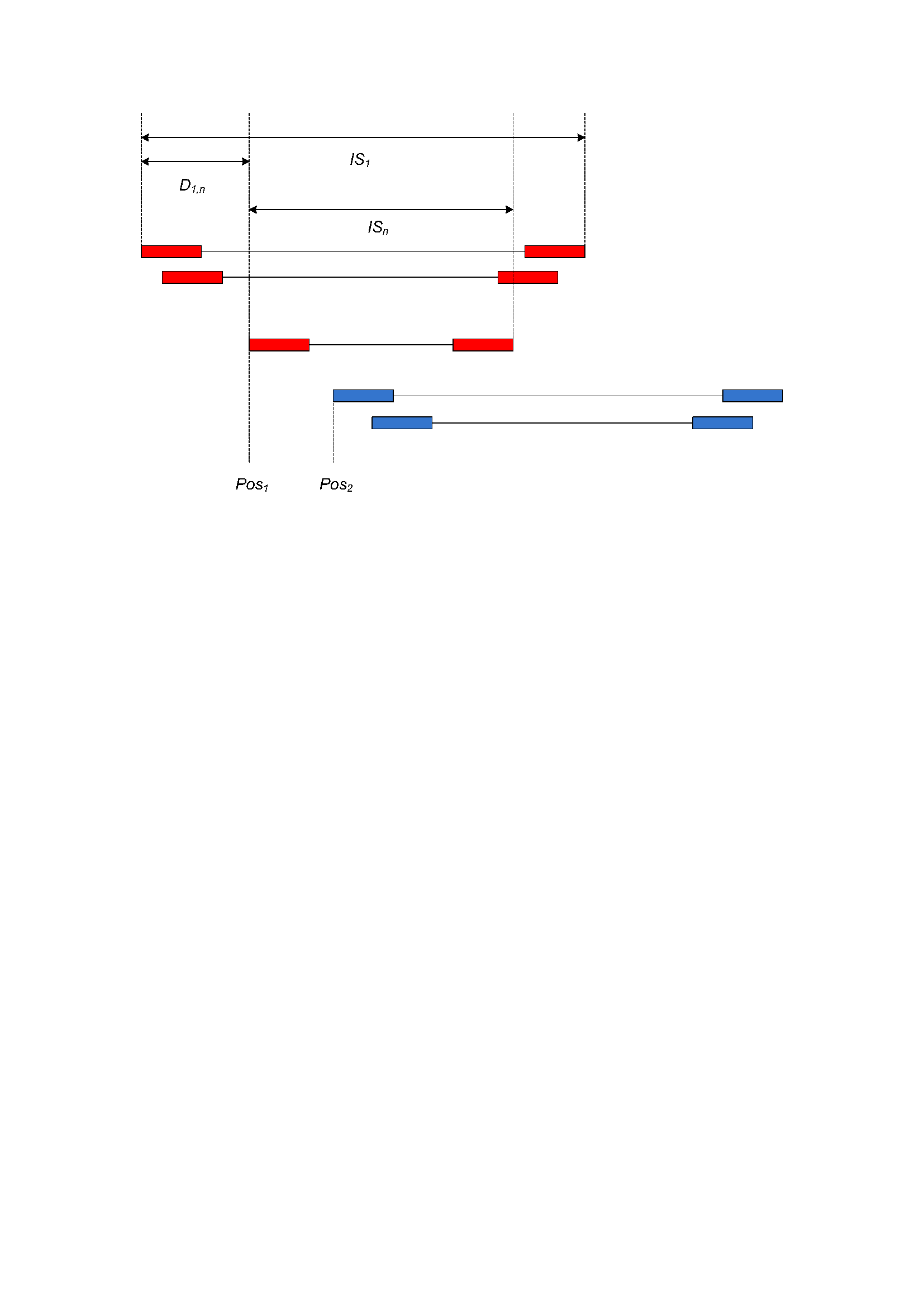

Supplement: Figure S8 — A synthetic example to illustrate additional requirements for candidate inversion clusters. Red reads are FF reads and blue ones are RR reads. All read pairs are ordered according to the start positions of their left mates. The insert size of the first pair is expected to be greater than or equal to the insert size of the last pair in the FF group, i.e., IS1> = ISn. The maximum distance (D1,n) between left mates of the FF read group is expected to be less than or equal to the mean insert size. If both the FF read group and the RR read group exist, the starting positions of all left mates of the FF group should be in front of any left mate in the RR group (i.e., Pos1< = Pos2). Similarly, one can derive their relationships for right mates and for the RR read group. (TIF) [file pone.0052881.s010.tif]

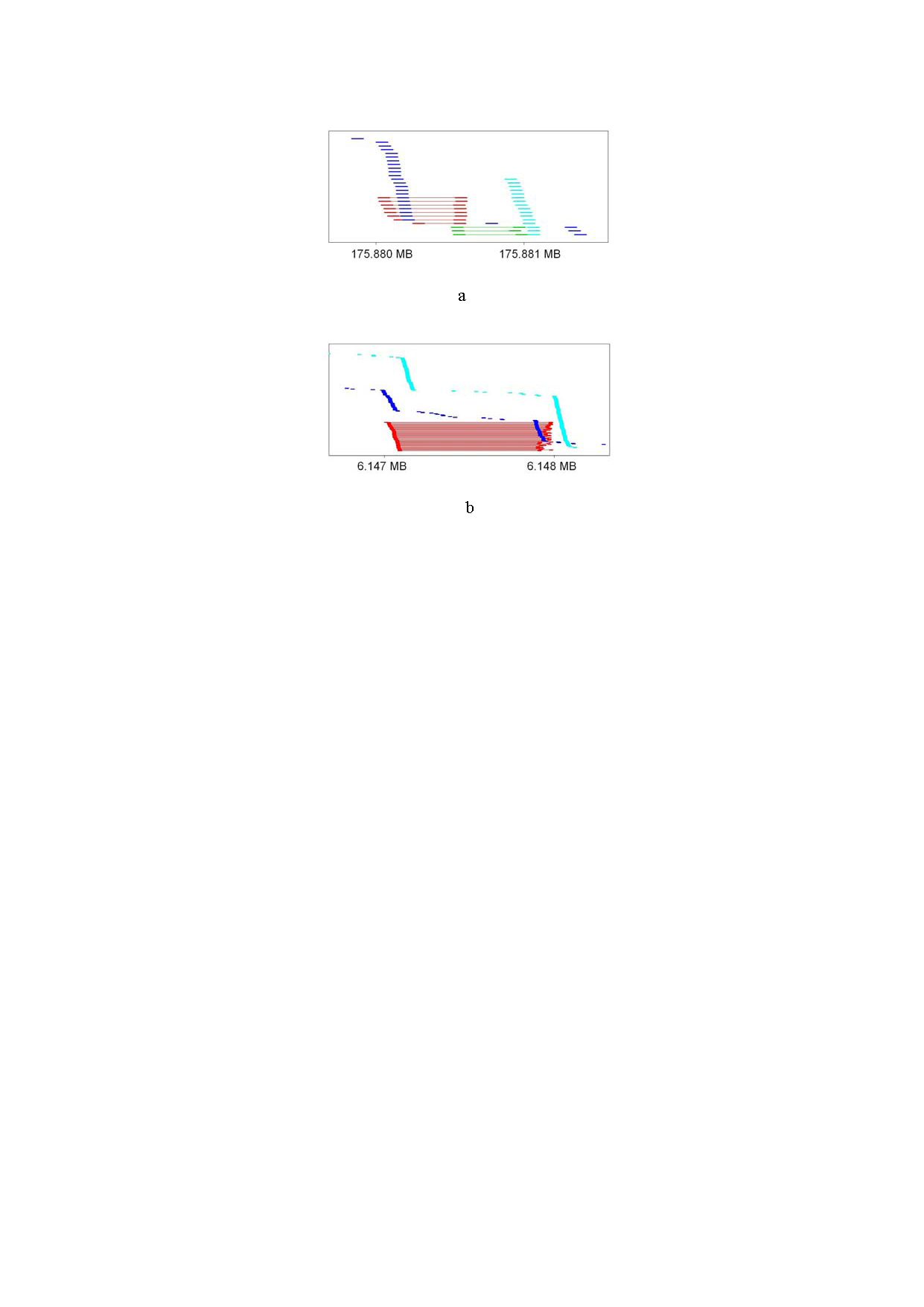

Supplement: Figure S9 — Examples of inversion events, one on chromosome 1 of NA18507 (a) and the other is on chromosome X of NA07340 (b). The FF discordant pairs are red and the RR pairs are green. Singleton reads are shown in dark blue and light blue, corresponding to forward reads and reverse reads, respectively. Note that (b) lacks RR discordant pairs. Singleton reads tend to accumulate near the inversion breakpoints because their mates do not map to the reference sequence due to potential overlap with the breakpoints. (TIF) [file pone.0052881.s011.tif]
